# Clinical Spatial Distribution of Aquaporin-1 in Camel Cornea Using Assistive AI Applications

**DOI:** 10.3390/vetsci13050425

**Published:** 2026-04-27

**Authors:** Liana Fericean, Ahmed Magdy, Reda Rashed, Khaled Shoghy, Adel Abdelkhalek, Ahmed Abdeen, Banatean-Dunea Ioan, Mihaela Ostan, Olga Rada, Mohamed Abdo

**Affiliations:** 1 Department of Biology and Plant Protection, Faculty of Agriculture, University of Life Sciences “King Michael I” from Timisoara, 300645 Timisoara, Romania; mihaelafericean@usvt.ro (L.F.); mihaela_ostan@usvt.ro (M.O.); olga_rada@usvt.ro (O.R.); 2 Department of Animal Histology and Anatomy, School of Veterinary Medicine, Badr University in Cairo (BUC), Cairo 11829, Egypt; ahmed.magdy@buc.edu.eg (A.M.); mohamed.abdo@vet.usc.edu.eg (M.A.); 3 Department of Anatomy and Embryology, Faculty of Veterinary Medicine, University of Sadat City, Sadat City 32897, Egypt; reda.rashed@vet.usc.edu.eg (R.R.); khaled.shoghy@vet.usc.edu.eg (K.S.); 4 Department of Food Safety, Hygiene and Technology, School of Veterinary Medicine, Badr University in Cairo (BUC), Cairo 11829, Egypt; adel.abdelkhalek@buc.edu.eg; 5 Department of Forensic Medicine and Toxicology, Faculty of Veterinary Medicine, Benha University, Toukh 13736, Egypt; ahmed.abdeen@fvtm.bu.edu.eg

**Keywords:** AQP1, cornea, one humped camel, immunohistochemistry

## Abstract

The cornea of the dromedary camel is essential for maintaining ocular clarity and protecting the eye in dry, dusty, and thermally stressful environments. Aquaporins are membrane channels that facilitate water transport, and AQP1 has been widely implicated in corneal fluid homeostasis in several species. The present work investigated, for the first time, the regional distribution of AQP1 in the camel cornea. Corneas collected from twelve healthy adult camels after slaughter were divided into nine anatomical regions: central (C), middle dorsal (MD), middle ventral (MV), middle nasal (MN), middle temporal (MT), peripheral dorsal (PD), peripheral ventral (PV), peripheral nasal (PN), and peripheral temporal (PT). Histological examination and immunohistochemistry were combined with digital morphometry to assess corneal layer thickness and AQP1 localization. AQP1 labeling was identified in the corneal epithelium, stromal keratocytes, and endothelium. Epithelial staining differed among regions and was most pronounced in the peripheral nasal region, whereas stromal keratocytes and endothelial cells showed strong and relatively uniform immunoreactivity. These findings indicate that AQP1 is broadly expressed in the camel cornea and likely contributes to regional control of hydration and tissue maintenance in an arid-adapted species.

## Simple Summary

Dromedary camels are continually exposed to heat, low humidity, airborne dust, and intense solar radiation, all of which challenge the integrity of the ocular surface. Because the cornea must remain transparent while maintaining a tightly regulated water balance, proteins involved in transcellular water movement are especially important in this species. In the present study, corneas from twelve clinically normal adult camels were examined to determine how aquaporin-1 (AQP1) is distributed across different corneal regions. Histology, immunohistochemistry, and computer-assisted morphometric measurements were used to compare epithelial, stromal, and endothelial features. AQP1 immunoreactivity was detected throughout the cornea, with particularly strong labeling in stromal keratocytes and endothelial cells and region-dependent epithelial staining. These findings support the view that the camel cornea has structural and molecular specializations that help preserve hydration under desert conditions. The observations may also be useful for comparative ophthalmology and for understanding corneal water regulation in other species.

## 1. Introduction

The global camel population is currently estimated at approximately 25–27 million individuals, including both one-humped (dromedary) and two-humped (Bactrian) camels, with numbers showing a recent upward trend. Among these, dromedary camels represent nearly 90% of the genus Camelus, making them the predominant species worldwide [1]. Widely distributed across the Middle East and Africa, the dromedary camel is considered one of the most valuable domestic animals in arid and semi-arid regions, primarily due to its remarkable ability to produce high-quality food under extreme environmental conditions [2].

Adaptation to harsh desert climates has led to the development of specialized anatomical and physiological features in the dromedary camel, particularly at the level of the eye. The ocular system plays a critical role in ensuring survival in environments characterized by intense solar radiation, dust exposure, and low humidity. Notably, the cornea functions as a protective barrier against external insults while also fulfilling essential optical and physiological roles. Structurally, it is a transparent and avascular tissue, properties that are fundamental for maintaining visual acuity [3,4].

Water homeostasis within the cornea is tightly regulated by a family of membrane proteins known as aquaporins (AQPs). In mammals, thirteen aquaporin isoforms (AQP0–AQP12) have been identified, primarily facilitating the transport of water, although some members are also capable of transporting small, uncharged solutes. Based on their permeability characteristics, AQPs are generally classified into two main groups: classical aquaporins, which are selective for water (including AQP0, AQP1, AQP2, AQP4, AQP5, AQP6, and AQP8), and aquaglyceroporins, which additionally allow the passage of molecules such as glycerol and hydrogen peroxide [5].

Among these, AQP1 has been shown to exhibit a highly conserved expression pattern across multiple species. It is predominantly localized in the corneal endothelium and has also been identified in stromal cells in a wide range of animals, including mice, pigs, cattle, goats, horses, dogs, cats, humans, rabbits, and rats [5]. Furthermore, both corneal endothelial cells and keratocytes express AQP1, highlighting its important role in maintaining corneal hydration, transparency, and structural integrity [6,7,8].

Despite the growing body of knowledge on aquaporin distribution in various species, detailed information regarding their spatial expression in the camel cornea remains limited. The cornea can be anatomically subdivided into nine distinct regions: peripheral dorsal (PD), peripheral ventral (PV), peripheral nasal (PN), peripheral temporal (PT), central (C), middle dorsal (MD), middle ventral (MV), and middle nasal (MN). Understanding the regional distribution of AQP1 across these areas may provide valuable insights into the adaptive mechanisms that support corneal function under extreme environmental stress. Therefore, the present study aims, for the first time, to investigate the spatial topographical expression of AQP1 in all corneal regions of the dromedary camel and to explore its potential role in adaptation to harsh desert conditions.

In parallel, recent advances in artificial intelligence (AI) have introduced new opportunities for enhancing diagnostic capabilities in veterinary medicine, particularly through automated image and physiological data analysis. AI-based approaches have demonstrated promising applications in companion animals such as dogs. However, their broader implementation in veterinary practice remains constrained by several factors, including the scarcity of large, well-annotated datasets, interspecies anatomical and pathological variability, and the significant financial and infrastructural limitations faced by many veterinary clinics [9,10]. Additionally, unlike human healthcare systems—where data collection is often standardized and supported by large institutions—veterinary data are frequently fragmented across multiple practices with heterogeneous recording systems [11]. These challenges highlight the need for robust, species-specific datasets and methodologies, particularly when integrating advanced analytical tools into veterinary research.

## 2. Materials and Methods

### 2.1. Experimental Samples

Corneal samples were obtained from twelve apparently healthy adult one-humped camels (*Camelus dromedarius*) at El-Basateen slaughterhouse, Cairo, Egypt, immediately after slaughter. The animals showed no gross ocular lesions at the time of collection. All procedures involving sampling, handling, and tissue processing were reviewed and approved by the institutional ethics committee of Sadat University (Ethical approval No. VUSC-002-1-26) and were conducted in accordance with applicable ethical guidelines (Table 1).

### 2.2. Tissue Preparation

**Figure 1:**
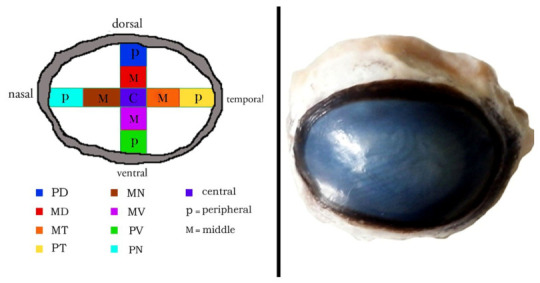
Topographic dissection of the camel cornea into specific anatomical segments. Areas identified include the central region (C) along with middle (MD, MV, MN, MT) and peripheral (PD, PV, PN, PT) sites, categorized by their dorsal, ventral, nasal, and temporal orientations.

Each freshly collected cornea was placed on a transparent support and subdivided with a sterile razor blade into nine anatomical regions representing the central, middle, and peripheral zones of the corneal surface. The analyzed regions were central (C), middle dorsal (MD), middle ventral (MV), middle nasal (MN), middle temporal (MT), peripheral dorsal (PD), peripheral ventral (PV), peripheral nasal (PN), and peripheral temporal (PT). To maintain orientation, the dorsal aspect was marked immediately after enucleation, and the tissue was then sectioned along the dorsal–ventral and nasal–temporal axes. This standardized sampling scheme was used for all specimens so that regional comparisons could be made consistently. Tissue fragments intended for histology and immunohistochemistry were immersed promptly in 10% neutral buffered formalin (Figure 1).

### 2.3. Histological Processing and Morphometric Analysis

Corneal samples were preserved in 10% neutral buffered formalin, followed by dehydration through a graded series of ethanol solutions, clarification in xylene, and subsequent embedding in paraffin wax. Tissue sections with a thickness of 4–5 μm were prepared using a rotary microtome (Leica RM2235, Leica Microsystems, Wetzlar, Germany) and mounted onto glass slides. Routine Hematoxylin and Eosin (H&E) staining was then performed according to established histological procedures. Sections were stained with hematoxylin and eosin (H&E) for morphometric analysis (*n* = 6 camels; 3 males, 3 females). Only sections exhibiting a strictly perpendicular orientation to the sectioning plane and a well-preserved trilaminar architecture (epithelium, stroma, and Descemet’s membrane) were included to ensure high-quality measurements. To avoid measurement bias, specimens with processing abnormalities such oblique sectioning, tissue folds, or mechanical breaks were excluded. Five non-consecutive portions (separated by at least 50 µm) were chosen for analysis from each biological replicate. To ensure that the mean thickness values obtained were representative of the whole corneal landscape, five non-overlapping microscopic areas were photographed at 100× magnification inside each slice.

### 2.4. Histological Staining

For general histological evaluation, paraffin sections were stained with hematoxylin and eosin according to standard laboratory procedures [12].

### 2.5. Immunohistochemistry of AQP1

Paraffin-embedded tissue sections were cut at a thickness of 4 μm, followed by deparaffinization in xylene and rehydration through a descending ethanol series (100%, 95%, and 75%). Antigen retrieval was performed using a heat-induced epitope retrieval (HIER) approach. Specifically, sections were treated with Cell Marque Trilogy solution and processed in a pressure cooker. In addition, citrate buffer (10 mM, pH 6.0) was used to enhance antigen unmasking, with sections heated in a water bath at 95–98 °C for 15–20 min and then allowed to cool gradually to room temperature. Following retrieval, sections were rinsed thoroughly in distilled water and phosphate-buffered saline (PBS).

Endogenous peroxidase activity was blocked by incubating the sections in 3% hydrogen peroxide. Subsequently, the sections were incubated for 60 min with a rabbit polyclonal anti-human AQP1 primary antibody (1:1000; catalog no. GB11310, Servicebio, Woburn city, MA, USA). After primary antibody incubation, sections were treated with a horseradish peroxidase (HRP)-linked secondary detection system (EnVision^TM^+ System-HRP, Dako, Denmark; catalog no. K4003) according to the manufacturer’s instructions for 15 min.

Immunoreactivity was visualized using a 3,3′-diaminobenzidine (DAB) chromogen system (DAB Substrate Kit, Vector Laboratories, Woburn city, MA, USA; catalog no. SK-4100). The working solution was freshly prepared by combining the chromogen and buffer components in a 1:1 ratio, and sufficient volume (approximately 200 µL per slide) was applied to cover each section. The reaction was monitored microscopically and stopped by rinsing in distilled water. Sections were then counterstained with hematoxylin, dehydrated through ascending grades of ethanol, cleared in xylene, and finally mounted with a permanent mounting medium and coverslipped [13].

To ensure staining specificity, negative control sections were processed in parallel using the same protocol but with omission of the primary antibody, which was replaced with PBS. Positive controls were also included in each staining run, consisting of kidney tissue known to express AQP1, particularly in proximal tubules and vascular endothelium. The presence of a distinct and specific staining pattern in these controls confirmed both antibody reactivity and the effectiveness of the detection system.

Although the primary antibody was raised against human AQP1, its use in camel tissue was considered appropriate due to the high degree of sequence conservation of AQP1 among mammalian species, supporting the likelihood of cross-reactivity.

All control and experimental sections were processed and evaluated under identical conditions to ensure consistency and reliability of the immunohistochemical results.

### 2.6. Quantification of AQP1 Immunoreactivity

AQP1 immunopositivity was quantitatively analyzed using ImageJ software Version 1.54s (National Institutes of Health, Washington, DC, USA) with the Trainable Weka Segmentation (TWS) plugin. Images for IHC quantification were obtained at 400× magnification (40× objective) to provide high-resolution data. Classifier Training: A Random Forest classifier was trained using a representative selection of corneal pictures. An expert manually annotated three different pixel classes: background, unstained tissue components, and AQP1-positive signals (DAB chromogen). Automated Inference: The classifier was applied to all 150 microscopic fields in the dataset (5 sections per animal × 5 fields per section; n = 6 camels) once it achieved optimum accuracy. For every field of vision, this produced binary segmentation masks that were consistent. Regional Quantification: Within the distinct biological areas of interest (ROIs) of the Epithelium, Stroma, and Endothelium, the Area Fraction (%) of AQP1 expression was calculated. An examiner blind to the names of the regional sample carried out all picture quantification and AI training to ensure objectivity.

### 2.7. Photomicroscopy and Qualitative Measurements

Histological and immunohistochemical sections were visualized using an Olympus [Insert Model, BX53] clinical microscope (Olympus Corp., Tokyo, Japan). High-resolution digital micrographs were captured using an Olympus [Insert Camera Model, DP27] digital camera system. To ensure data consistency, all images were acquired under fixed exposure times, gain settings, and white balance using Olympus cell Sens [Insert Version, Standard 1.18] software.

### 2.8. Measurement of Corneal Layer Thickness

Corneal layer thicknesses were measured on H&E-stained sections for the epithelium, stroma, and Descemet’s membrane. Before measurement, the imaging system was calibrated with a stage micrometer. For each animal, three non-consecutive sections were assessed, and several randomly selected sites within each predefined corneal region were measured. Thickness values were obtained in ImageJ by drawing lines perpendicular to the corresponding anatomical boundaries. Only areas with proper orientation and without obvious sectioning artifacts were included, and the mean value for each region was used in the statistical comparisons.

### 2.9. AI-Assisted Digital Image Analysis

Micrographs were captured using a light microscope equipped with a digital camera under strictly uniform lighting and magnification to ensure data consistency. Quantitative analysis was performed using the ImageJ software (National Institutes of Health, USA) integrated with the Trainable Weka Segmentation (TWS) plugin, an assistive Artificial Intelligence (AI) framework. Unlike conventional global thresholding, this study employed a supervised machine learning approach using a Random Forest classifier. The AI was trained on representative corneal sections to distinguish AQP1-positive signals from the background based on a high-dimensional feature space, including Gaussian blur, Sobel filters, and Hessian membranes. This AI-driven segmentation provided objective quantification of the immunostained area fraction across the pre-defined regions of interest (Epithelium, Stroma, and Endothelium). To ensure reproducibility and validate the AI’s performance, automated results were periodically cross-referenced with expert manual annotations, maintaining standardized protocols across all biological replicates.

### 2.10. Machine Learning-Assisted Image Segmentation

An Artificial Intelligence (AI) framework was created using supervised machine learning utilizing the Trainable Weka Segmentation (TWS) platform to guarantee high-precision quantification and reduce inter-observer variability.

#### 2.10.1. Training Protocol and Class Definition

To calibrate the classifier, a representative training library was created using the picture dataset. Pixels were manually annotated by experts into three different ground-truth classes: 1. AQP1-positive: Chromogenic signals specific to diaminobenzidine (DAB). 2. Background: Slide artifacts and luminal spaces. 3. Non-specific Tissue: Cellular components that are either unstained or counterstained.

#### 2.10.2. Feature Engineering and Architecture

A Random Forest classifier was used by the segmentation engine because of its strong performance in challenging histology settings. To advance beyond basic intensity thresholding, the classifier was set up utilizing a multi-dimensional feature space. Important training components included: 1. Intensity and Space Entropy, variance, and mean are characteristics. 2. Edge detection: boundary definition using Sobel and Hessian filters. 3. Texture and Scale: To capture fine protein distribution patterns, Difference of Gaussians (DoG) and multi-scale Gaussian filters were used.

#### 2.10.3. Automated Segmentation and Quantification

The trained classifier was applied to the complete dataset for automated, pixel-by-pixel inference once it had been optimized. High-resolution probability maps were produced as a result, and these were transformed into binary masks such that quantitative measurements could be extracted. Within certain Regions of Interest (ROIs) that corresponded to the epithelium, stroma, and endothelium, the Area Fraction (%) and Mean Integrated Density of AQP1 were calculated.

#### 2.10.4. Validation of Segmentation

An expert histologist’s hand segmentations of a subset of 20 figures were compared with the AI-generated findings to verify AI’s performance. The reliability and impartiality of the AI application in measuring camel corneal AQP1 were confirmed by the strong correlation (Pearson’s r > 0.90).

### 2.11. Statistical Analysis

Quantitative data were analyzed using IBM SPSS Statistics (Version 28.0; IBM Corp., Armonk, NY, USA) [14,15]. To ensure biological independence and prevent pseudoreplication, the individual animal (n = 12) was defined as the primary statistical unit. The study design utilized two distinct cohorts:

1. Morphometric Group (n = 6; 3 males, 3 females): Used for H&E-based thickness measurements.
2. Immunohistochemical (IHC) Group (n = 6; 3 males, 3 females): Used for AQP1 expression analysis.

For each cornea, multiple histological sections and non-overlapping fields of view were measured to generate a single representative mean per animal. Data distribution and homogeneity of variance were verified using the Shapiro–Wilk and Levene’s tests, respectively. Significant differences in corneal thickness (epithelium, stroma, and Descemet’s membrane) and AQP1 area fraction (epithelium, stroma, and endothelium) were determined using One-way Analysis of Variance (ANOVA). Where significant main effects were detected, Tukey’s Honest Significant Difference (HSD) post hoc test was applied for pairwise comparisons between the three corneal layers. All numerical results are expressed as Mean ± Standard Deviation (SD), and statistical significance was pre-defined at *p* < 0.05.

## 3. Results

### 3.1. Histological Staining

#### 3.1.1. Epithelium

**Figure 2:**
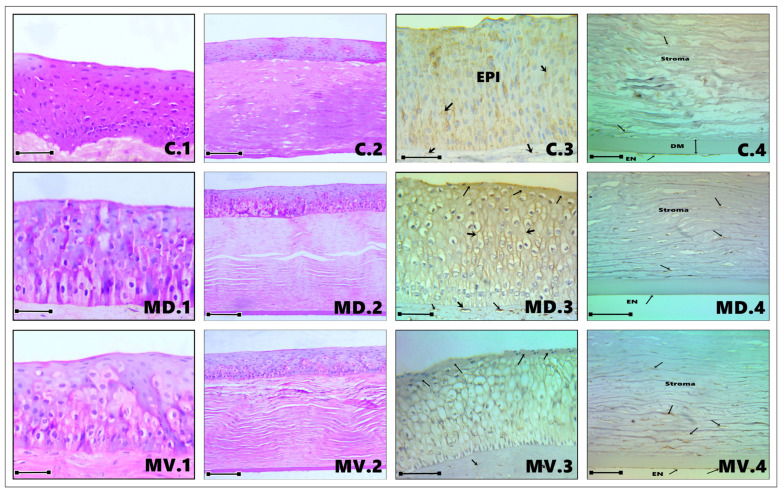
Representative photomicrographs of the camel cornea. Panels (C1, MD1, MV1) show H&E-stained sections illustrating epithelial thickness in the central (C), middle dorsal (MD), and middle ventral (MV) regions (scale bar: 100 µm). Panels (C2, MD2, MV2) depict the stromal layer and Descemet’s membrane in the same regions following H&E staining. Panels (C3, MD3, MV3) demonstrate AQP1 immunoreactivity within the corneal epithelium and keratocytes of the anterior stroma, with variable staining intensity across regions (black arrows; scale bar: 50 µm). Panels (C4, MD4, MV4) show AQP1 localization in the posterior stroma and endothelium, where immunostaining is primarily confined to keratocytes and endothelial cells (black arrows; scale bar: 50 µm).

**Figure 3:**
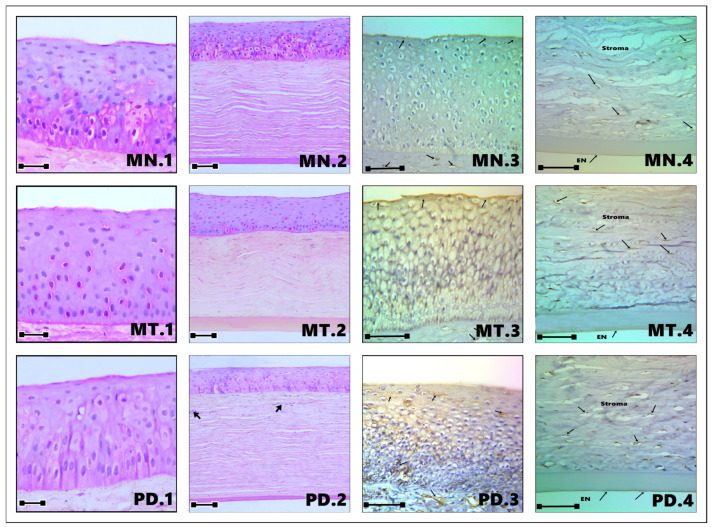
Representative photomicrographs of the camel cornea from the middle nasal (MN), middle temporal (MT), and peripheral dorsal (PD) regions. Panels (MN1, MT1, PD1) show H&E-stained sections illustrating epithelial thickness in the corresponding regions. In addition, vascular structures are visible in the peripheral dorsal region (PD2), likely associated with the limbal area (black arrows; scale bar: 100 µm). Panels (MN2, MT2, PD2) demonstrate the stromal layer and Descemet’s membrane in these regions following H&E staining. Panels (MN3, MT3, PD3) reveal AQP1 immunoreactivity within the corneal epithelium and keratocytes of the anterior stroma, with regional variation in staining intensity (black arrows; scale bar: 50 µm). Panels (MN4, MT4, PD4) illustrate AQP1 localization in the posterior stroma and endothelium, where staining is predominantly confined to keratocytes and endothelial cells (black arrows; scale bar: 50 µm).

**Figure 4:**
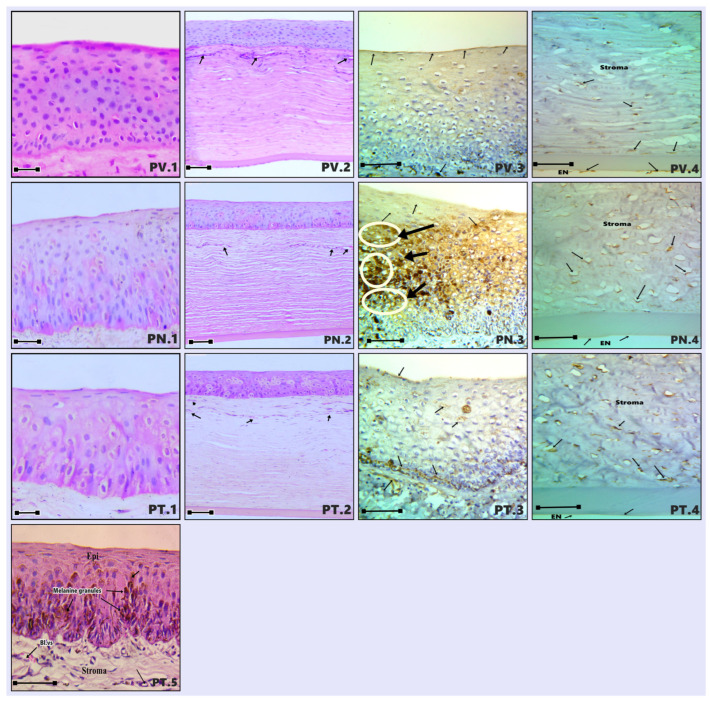
Representative photomicrographs of the camel cornea from the peripheral ventral (PV), peripheral nasal (PN), and peripheral temporal (PT) regions. Panels (PV1, PN1, PT1) show H&E-stained sections illustrating epithelial thickness in the respective regions (scale bar: 100 µm). Vascular structures are evident in the peripheral areas (PV2, PN2, PT2), likely corresponding to extensions of the limbal vasculature (black arrows). Panels (PV2, PN2, PT2) further demonstrate the stromal layer and Descemet’s membrane following H&E staining. Panels (PV3, PN3, PT3) display AQP1 immunoreactivity within the corneal epithelium and keratocytes of the anterior stroma, with noticeable regional differences in staining intensity (black arrows; scale bar: 50 µm). The strongest epithelial expression of AQP1 was observed in the peripheral nasal region (PN3), highlighted by white circles. Panels (PV4, PN4, PT4) illustrate AQP1 localization in the posterior stroma and endothelium, where staining is primarily confined to keratocytes and endothelial cells (black arrows; scale bar: 50 µm). Additionally, panel (PT5) shows the presence of brown melanin granules within the peripheral temporal region (black arrows; scale bar: 50 µm).

The cornea was observed as a thin, transparent, and completely avascular structure (Figure 1), composed of distinct histological layers, including the epithelium, Bowman’s membrane, stroma, Descemet’s membrane, and endothelium. These layers were consistently identified across all examined regions (Figure 2: C1, MD1, MV1; Figure 3: MN1, MT1, PD1; Figure 4: PV1, PN1, PT1).

The anterior corneal epithelium consisted of a non-keratinized stratified squamous epithelium exhibiting a multilayered organization, typically comprising approximately 10–12 cell layers. The intermediate zone was formed by 6–8 layers of polyhedral cells characterized by eosinophilic cytoplasm and centrally located, round, intensely stained nuclei. In contrast, the basal layer consisted of 2–3 layers of flattened cells displaying elongated, densely stained nuclei and strongly eosinophilic cytoplasm (Figure 2, Figure 3 and Figure 4).

Notably, variable deposits of brown melanin granules were detected within the epithelial cells of the peripheral corneal regions, particularly in the nasal and temporal areas (Figure 4: PN1, PT5).

**Figure 5:**
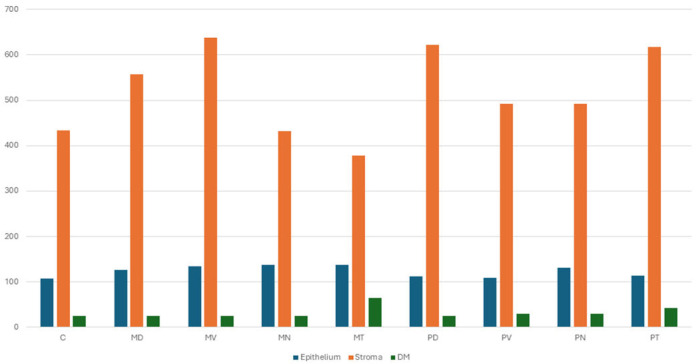
Morphometric analysis of camel corneal layers Mean Thickness (µm)—including the epithelium, stroma, and Descemet’s membrane—was performed across all examined regions: central (C), middle dorsal (MD), middle nasal (MN), middle temporal (MT), middle ventral (MV), peripheral dorsal (PD), peripheral nasal (PN), peripheral temporal (PT), and peripheral ventral (PV). The results indicated regional variation in layer thickness, with the middle temporal (MT) region exhibiting the greatest thickness of Descemet’s membrane, while the middle ventral (MV) region showed the highest stromal thickness.

Morphometric analysis (Table 2; Figure 5) demonstrated clear regional differences in epithelial thickness. The middle corneal regions (MD, MV, MN, and MT) exhibited the greatest thickness values, ranging approximately 11–22% higher than those recorded in the central and peripheral regions. The maximum thickness was observed in the middle temporal (MT) region (137.71 µm), whereas the central region presented the lowest value (107.09 µm). Overall, the epithelium was consistently thickest in the middle corneal zones, particularly in the order: MT, MN, MV, and MD (Figure 2: MD1, MV1; Figure 3: MT1, MN1).

The basement membrane was clearly delineated in several sections, particularly in the middle temporal and middle nasal regions (Figure 3: MT1, MT2, MN2), where it appeared as a well-defined interface between the epithelium and the underlying stroma.

#### 3.1.2. Stroma

The corneal stroma represented the most substantial component of the corneal structure, accounting for approximately 90% of its total thickness. It was characterized by a dense organization of collagen fibers interspersed with fibroblastic cells (keratocytes), which were distributed between the lamellae of collagen fibrils (Figure 2: C2, MD2, MV2; Figure 3: MN2, MT2, PD2; Figure 4: PV2, PN2, PT2). The extracellular matrix exhibited a highly ordered arrangement of parallel collagen fibrils of uniform diameter, closely packed to maintain corneal transparency. Histologically, the stromal layer displayed a typical eosinophilic appearance, ranging from pink to magenta following H&E staining.

Although the cornea is physiologically avascular, occasional vascular profiles were observed in the peripheral regions, particularly near the corneal–limbal junction (Figure 3: PD2; Figure 4: PV2, PN2, PT2). These vessels likely correspond to extensions of the limbal vascular network rather than true stromal vascularization.

Morphometric data (Table 3; Figure 5) confirmed that the stroma constituted the largest proportion of corneal thickness across all regions. The highest stromal thickness values were recorded in the middle ventral (MV) region (637.22 µm) and the peripheral dorsal (PD) region (622.22 µm), whereas the middle temporal (MT) region exhibited the lowest measured thickness (378.33 µm).

#### 3.1.3. Descemet’s Membrane (DM)

The Descemet’s membrane was identified as a distinct, homogeneous layer situated between the stroma and the endothelium (Figure 2: C2, MD2, MV2; Figure 3: MN2, MT2, PD2; Figure 4: PV2, PN2, PT2). Histologically, it appeared as a well-defined, eosinophilic structure with an amorphous composition. This layer was continuous and relatively thick compared to other corneal components. Morphometric evaluation (Table 4; Figure 5) indicated that Descemet’s membrane exhibited a generally consistent thickness across most corneal regions, ranging approximately between 25 and 30 µm. However, a notable regional variation was observed, with the middle temporal (MT) area displaying a substantially greater thickness (63.89 µm), representing the highest recorded value among all examined regions.

#### 3.1.4. Endothelium

The corneal endothelium consisted of a single layer of flattened cells lining the posterior surface of the cornea. Cell borders were not sharply demarcated in all sections, but the endothelial lining was consistently recognizable in every analyzed region.

### 3.2. Aquaporin-1 Immuno-Expression

**Figure 6:**
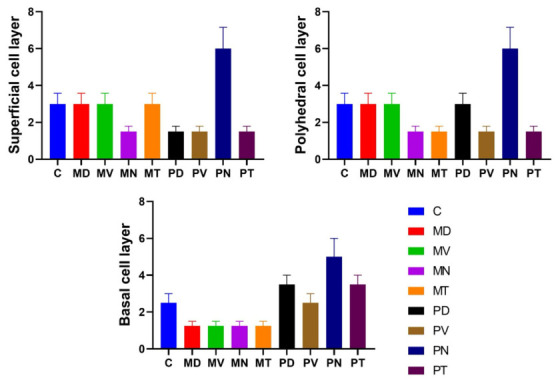
Immunohistochemical localization of AQP1 in camel corneal epithelium across different cellular layers, including superficial, intermediate (polyhedral), and basal cells. The columns represent the relative expression levels of AQP1 in the following corneal regions according to Area Fraction (%): central (C), middle dorsal (MD), middle nasal (MN), middle temporal (MT), middle ventral (MV), peripheral dorsal (PD), peripheral nasal (PN), peripheral temporal (PT), and peripheral ventral (PV). Data are presented as Mean ± SD (n = 6). Different superscript letters above bars indicate statistically significant differences between groups (One-way ANOVA followed by Tukey’s post hoc test, *p* < 0.05).

**Figure 7:**
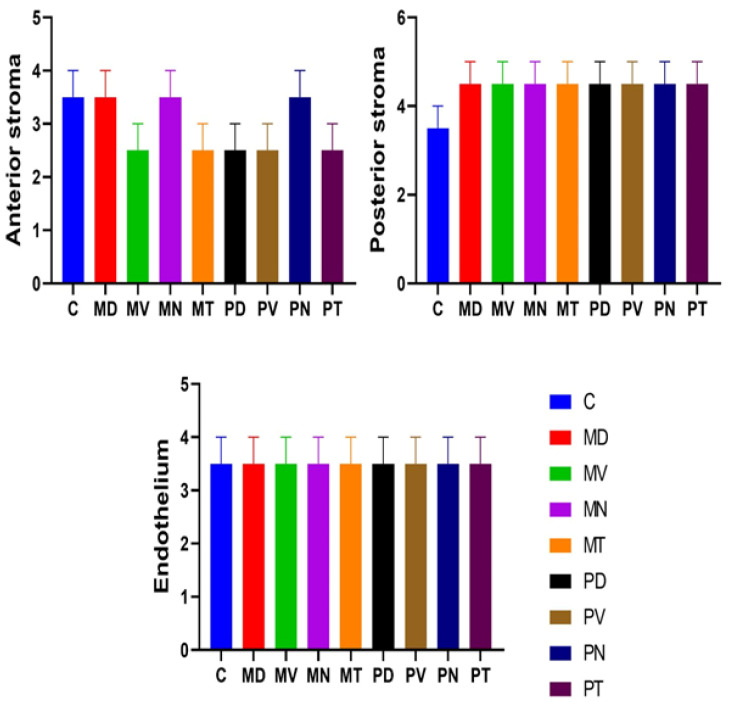
Immunohistochemical distribution of AQP1 in the camel cornea, including the anterior and posterior stromal regions as well as the endothelium. The columns illustrate the relative expression levels of AQP1 across different corneal regions according to Area Fraction (%): central (C), middle dorsal (MD), middle nasal (MN), middle temporal (MT), middle ventral (MV), peripheral dorsal (PD), peripheral nasal (PN), peripheral temporal (PT), and peripheral ventral (PV). Data are presented as Mean ± SD (n = 6). Different superscript letters above bars indicate statistically significant differences between groups (One-way ANOVA followed by Tukey’s post hoc test, *p* < 0.05).

The immunolocalization of aquaporin-1 (AQP1) was evaluated across the three principal corneal layers—epithelium, stroma, and endothelium—as illustrated in Figure 6 and Figure 7. AQP1 expression was detected in all layers, although its distribution and staining intensity varied depending on the corneal region and cellular component.

Within the corneal epithelium, AQP1 exhibited a positive immunoreaction in all examined regions and across all epithelial layers (Figure 2: C3, MD3, MV3; Figure 3: MN3, MT3, PD3; Figure 4: PV3, PN3, PT3). However, differences in staining intensity were observed among the superficial, intermediate (polyhedral), and basal cell layers (Figure 6). The peripheral nasal (PN) region demonstrated the most intense immunoreactivity across all epithelial layers. In contrast, the central (C), middle dorsal (MD), middle ventral (MV), and middle temporal (MT) regions showed moderate and relatively uniform staining, particularly within the superficial epithelial cells. Lower staining intensity in the superficial layer was noted in the MN, PD, PV, and PT regions.

A similar pattern was observed in the intermediate (polyhedral) cell layer, where moderate AQP1 expression was evident in the C, MD, MV, and PD regions, while comparatively weaker staining was detected in MN, MT, PV, and PT (Figure 6). In the basal layer, AQP1 immunoreactivity was present across all corneal regions, although variations in intensity were less pronounced compared to the superficial and intermediate layers.

According to Figure 6, quantitative AI-assisted analysis revealed significant regional variations in AQP1 expression within the corneal epithelium. In the superficial and polyhedral cell layers, the PN region exhibited the highest level of immunopositivity (6.02pm 1.1%), which was significantly greater than all other anatomical regions examined (F (8, 45) = 14.32, *p* < 0.001; Tukey’s HSD, *p* < 0.05). In contrast, the basal cell layer showed a more heterogeneous distribution. While the PN region remained elevated (5.1 pm 0.9%), it was followed closely by the PD region (3.5 pm 0.5%). The lowest expression levels were consistently observed in the MN and MT regions across all epithelial strata, representing a statistically significant decrease compared to the peripheral zones (*p* < 0.01).

**Figure 8:**
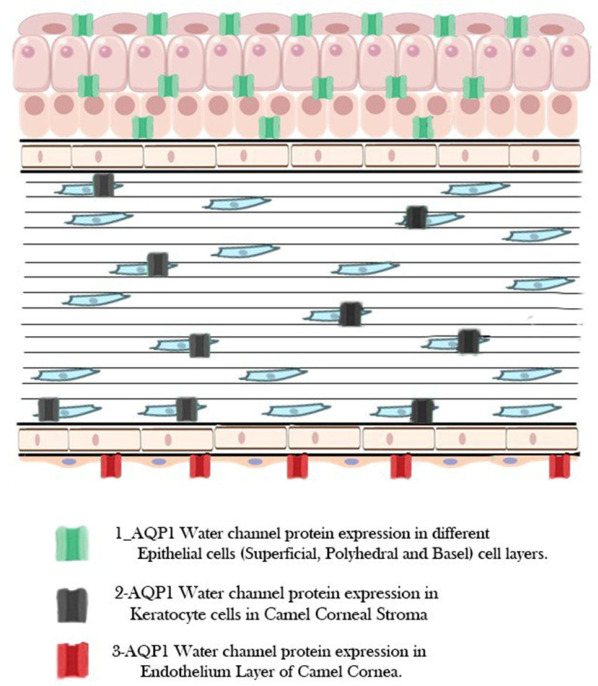
Proposed model for the spatial distribution of AQP1 water channels in the camel cornea in the three corneal layers, epithelium, stroma and endothelium. The green color shows AQP1 localization in the different corneal epithelial cell layers; superficial, polyhedral, and basal cell layers. The black color shows localization of AQP1 in keratocyte cells of stroma, while the red color clarifies the localization of AQP1 in corneal endothelium.

**Figure 9:**
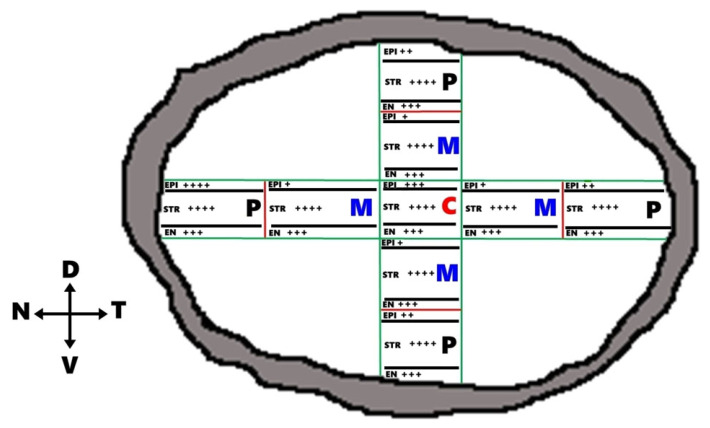
Topographical map of AQP1 distribution across the nine corneal regions. The schematic represents the regional intensity of AQP1 expression in the epithelium (EPI), stroma (STR), and endothelium (EN) of the camel cornea. The AI-generated Area Fraction (AF %) data: (+) = Weak expression (AF < 2%), (++) = Moderate expression (AF = 2–4%), (+++) = Strong expression (AF = 4–6%) and (++++) = Very strong expression (AF > 6%).

In the corneal stroma, AQP1 expression was restricted to keratocytes, with no detectable staining observed in the collagen fibers (Figure 2: C4, MD4, MV4; Figure 3: MN4, MT4, PD4; Figure 4: PV4, PN4, PT4). Quantitative analysis (Figure 7, Figure 8 and Figure 9) revealed regional differences in AQP1 expression between the anterior and posterior stromal compartments. Specifically, higher expression levels in the anterior stroma were identified in the C, MD, MN, and PN regions, whereas the MV, MT, PD, PV, and PT regions exhibited comparatively lower and more uniform expression. In contrast, AQP1 expression in the posterior stroma was generally consistent across all regions, except for the central area, which showed slight variation.

According to Figure 7, the distribution of AQP1 in the corneal stroma showed distinct patterns between the anterior and posterior zones. In the anterior stroma, expression was significantly higher in the C, MD, and MN regions compared to the MV and MT zones (F(8, 45) = 5.88, *p* = 0.002).Conversely, the posterior stroma exhibited a remarkably uniform distribution across most regions, with the exception of the Central zone, which showed a slight but significant reduction in AQP1 density (3.5 pm 0.5%) compared to the peripheral and mid-peripheral regions (4.5 pm 0.6%; *p* < 0.05).

The corneal endothelium demonstrated strong and consistent AQP1 immunoreactivity across all examined regions (Figure 2: C4, MD4, MV4; Figure 3: MN4, MT4, PD4; Figure 4: PV4, PN4, PT4). Statistical analysis (Figure 7, Figure 8 and Figure 9) confirmed that both the intensity and distribution of AQP1 expression in the endothelial layer were uniform, with no significant regional differences detected. The endothelium displayed a completely homogenous expression profile (3.5 pm 0.5%), with no statistically significant differences observed between any of the nine topographical regions (F (8, 45) = 0.12, *p* = 0.98).

## 4. Discussion

### 4.1. Histological Staining

The corneal epithelium constitutes a critical interface between the tear film and the underlying stroma, playing an essential role in maintaining a smooth optical surface and preserving corneal transparency and refractive function [16]. Disruption of this epithelial barrier may facilitate the entrance of pathogens, including bacteria and viruses, into the stromal layer, where infections can initiate and propagate. In particular, viral replication often begins within compromised epithelial cells, highlighting the importance of epithelial integrity in ocular defense. Additionally, epithelial wound healing involves rapid migration of cells from the margins of the defect; however, stable adhesion between the epithelium and stroma is not fully restored until a new basement membrane is formed. In its absence, recurrent epithelial erosions may occur.

Previous investigations in wild ruminants have described the cornea as a multilayered structure composed of a stratified squamous non-keratinized epithelium, a stromal layer (substantia propria), Descemet’s membrane, and a posterior endothelial layer [17]. In the present study, the dromedary camel cornea exhibited comparable structural organization. Notably, an anterior limiting layer, corresponding to Bowman’s membrane, was also identified beneath the epithelial layer. Although some reports describe the camel cornea as consisting of four principal layers, our observations support the presence of Bowman’s layer as a distinct structural component, in agreement with previous studies [18,19,20,21,22,23]. In contrast, in several other domestic species, the cornea is commonly described as comprising five layers, including a well-defined Bowman’s membrane [21,24,25,26].

The identification of Bowman’s layer in the camel cornea may have functional significance. It has been suggested that this structure contributes to the regulation of stromal hydration and resistance to swelling, particularly in the anterior stroma [27]. This feature may represent an adaptive mechanism enhancing corneal stability under extreme environmental conditions. Comparative studies further indicate that corneal morphology and thickness vary among species, reflecting differences in ecological adaptation and functional requirements [28].

In the present study, the corneal epithelium accounted for approximately 36% of the total corneal thickness, with greater thickness observed in the middle regions compared to the peripheral areas. This regional variation may reflect differential functional demands, as the central and middle zones are more directly exposed to environmental stressors and therefore require enhanced protection. In contrast, peripheral regions benefit from the presence of melanin pigmentation, which provides additional shielding. A thicker epithelium may also contribute to reducing stromal dehydration by limiting excessive water loss from deeper layers, particularly under high-temperature conditions. Furthermore, basal epithelial cells in the camel cornea appeared larger than those reported in humans and cattle, suggesting that environmental factors may influence epithelial morphology [19].

Comparative data indicate variability in corneal thickness among domestic species, with cattle exhibiting relatively thick corneas, followed by pigs and donkeys, while thinner corneas are reported in sheep, camels, goats, and buffalo [29]. However, the present findings suggest that the camel cornea is relatively thick compared to some species, particularly due to the substantial contribution of both the stroma and epithelium. This observation is consistent with previous reports highlighting the importance of increased corneal thickness as an adaptive feature in arid environments [28]. The cornea serves both protective and optical functions, and its transparency and refractive properties are essential for vision. Consequently, structural adaptations in the camel cornea may enhance its resistance to environmental stress while maintaining optical performance.

The presence of melanin granules within the peripheral corneal epithelium was also confirmed in this study, in agreement with previous reports in dromedary camels [3,20,26,30]. These pigments likely serve a protective role by absorbing excess light and reducing the damaging effects of ultraviolet (UV) radiation [20]. Such pigmentation may be particularly advantageous in desert environments, where intense solar radiation and glare are common [3].

The corneal stroma, which constitutes approximately 90% of total corneal thickness in most mammals, is composed of regularly arranged collagen lamellae that provide both transparency and mechanical strength [31]. In the present study, the stromal collagen fibers were organized in a lamellar pattern, contributing to the structural integrity and optical clarity of the cornea. These findings are consistent with previous studies in camels and other species [3,17,32]. Notably, the stromal thickness was greatest in the middle ventral region, where collagen bundles appeared dense, wavy, and well-organized, suggesting enhanced mechanical support in this area [19,33].

Descemet’s membrane was identified as a distinct layer located between the stroma and the endothelium, functioning as the basement membrane for endothelial cells. This layer plays a key role in maintaining corneal transparency and structural stability, as previously described in other species [17,19]. In the current study, Descemet’s membrane exhibited a relatively consistent thickness across most regions (25–30 µm), with a marked increase in the middle temporal region. This observation aligns with previous findings indicating that Descemet’s membrane in camels is thicker compared to other species [3]. Such structural reinforcement may enhance resistance to environmental stressors such as wind, dust, and mechanical impact.

The corneal endothelium consisted of a single layer of flattened (simple squamous) cells, consistent with observations in other animal species [22,28,34]. This layer is responsible for regulating fluid transport between the stroma and the aqueous humor, thereby maintaining corneal hydration and transparency [16]. Dysfunction of the endothelium may result in stromal edema due to the accumulation of fluid, leading to loss of corneal clarity. Additionally, the endothelium is capable of producing Descemet’s membrane, particularly in response to injury, further emphasizing its role in maintaining corneal integrity.

Overall, the structural characteristics observed in the camel cornea reflect a series of adaptive features that support survival in arid and harsh environments. These findings suggest that the camel cornea may serve as a valuable model for studying the effects of environmental stress on ocular tissues, with potential implications for both veterinary and comparative ophthalmology.

### 4.2. Aquaporin-1 Immuno-Expression

#### 4.2.1. Epithelium

The cornea serves as both a structural and functional interface with the external environment, requiring a precise balance between transparency, refractive capacity, and resistance to environmental stressors. Much of the current understanding of corneal structure and physiology is derived from studies using laboratory animals, particularly rodents, which are commonly used as models for human ocular research [13,35]. In agreement with previous findings, the present study in the dromedary camel indicates that aquaporins (AQPs) play a key role in maintaining corneal hydration and homeostasis under extreme environmental conditions [36,37].

Aquaporins are integral membrane proteins that facilitate rapid water movement across cell membranes and contribute to the regulation of cell volume and intercellular fluid exchange. These proteins are widely distributed across different biological systems, including animals, plants, and microorganisms [38]. Among them, AQP1 is one of the most extensively characterized isoforms, both structurally and functionally [39]. It has been implicated in a variety of physiological processes, including tear production and cerebrospinal fluid formation, although its exact role in ocular surface physiology and disease remains incompletely understood [40,41,42]. Additionally, AQP1 has been investigated in pathological conditions such as brain edema, where modulation of its activity may have therapeutic potential [41].

Previous studies have reported a relatively conserved pattern of AQP1 expression across mammalian species, with predominant localization in the corneal endothelium and lower expression in stromal keratocytes, while other aquaporins such as AQP3 and AQP5 are typically associated with the corneal epithelium [5]. However, the present study demonstrated, for the first time, the presence of AQP1 immunoreactivity within the corneal epithelium of the dromedary camel. This finding contrasts with observations in other domestic species [13] and may reflect a species-specific adaptation to arid environments, where efficient regulation of water content is essential for maintaining corneal function.

In mammals, thirteen aquaporin isoforms (AQP0–AQP12) have been identified, with some facilitating water transport exclusively, while others also permit the passage of small neutral solutes. Based on these properties, AQPs are broadly classified into classical aquaporins and aquaglyceroporins [5]. Continuous regulation of water and solute movement is critical for preserving corneal transparency, which depends on tightly controlled stromal hydration.

AQP1 plays a central role in corneal fluid dynamics. In the endothelium, it contributes to water transport from the stroma toward the anterior chamber, thereby preventing fluid accumulation and maintaining tissue clarity [6,43,44,45,46]. In stromal keratocytes, AQP1 has been associated with the regulation of cellular volume in response to changes in hydration status [6,8]. Furthermore, it has been suggested that AQP1 facilitates keratocyte migration during wound healing by enabling localized water influx at the leading edge of migrating cells [47]. In addition, AQP1 may contribute to the maintenance of limbal stem cell niches, supporting epithelial renewal processes [44].

In the present study, AQP1 was detected across all layers of the corneal epithelium, although staining intensity varied between regions. The highest expression was observed in the peripheral nasal region, while other regions showed moderate or lower levels of immunoreactivity. This heterogeneous distribution suggests functional specialization within the corneal epithelium, where different regions may respond differently to environmental stress. Peripheral areas, particularly those associated with the limbus, are known to serve as a reservoir for epithelial regeneration [31], and increased AQP1 expression in these regions may support both protective and regenerative functions.

Although central and middle corneal regions may experience relatively stable conditions, AQP1 expression in these areas remains important for maintaining hydration, particularly at the epithelial surface exposed to environmental factors. Overall, the observed gradient in AQP1 distribution supports the concept that the camel cornea exhibits region-specific adaptations, enabling it to maintain structural integrity and function under harsh desert conditions.

#### 4.2.2. Stroma

Keratocytes demonstrated positive immunoexpression of AQP1 across all stromal regions, although variations in expression intensity were observed between different corneal areas. This finding is consistent with previous reports indicating that AQP1 expression may vary among species and tissues [13]. It has been suggested that AQP1 contributes to the regulation of keratocyte volume in response to fluctuations in corneal hydration [6,8].

In addition, AQP1 may play an important role in corneal wound healing. It has been proposed that aquaporins facilitate keratocyte migration by enabling rapid water flux, allowing cells to move efficiently through the dense stromal matrix and narrow extracellular spaces. This mechanism supports previous findings on the involvement of aquaporins in corneal repair following injury [48].

In the present study, AQP1 expression within the stroma was localized exclusively to keratocytes, while no immunoreactivity was detected in the collagen fibers. Given that keratocytes are the principal cellular component of the stroma, their strong AQP1 expression suggests a key role in regulating stromal hydration and maintaining tissue transparency. The corneal stroma accounts for approximately 90% of total corneal thickness, and precise control of its water content is essential for preserving optical clarity.

This function is particularly important in desert environments, where maintaining appropriate hydration levels is critical to prevent stromal edema and subsequent visual impairment. Keratocytes, through their high AQP1 expression, likely act as regulators of fluid balance within the stroma, ensuring optimal hydration under extreme conditions.

The absence of AQP1 expression in collagen fibers further indicates that water transport within the stroma is mediated primarily by cellular elements rather than the extracellular matrix. While collagen fibrils provide structural organization and mechanical strength, they do not appear to participate directly in fluid regulation. These findings emphasize the central role of keratocytes in maintaining stromal homeostasis and corneal

#### 4.2.3. Endothelium

In the present study, AQP1 was consistently detected in the corneal endothelium of all examined camel samples, in agreement with previous reports describing similar expression patterns across different mammalian species, including domestic animals [8,13,37]. The presence of AQP1 in this layer highlights its essential role in maintaining corneal transparency by regulating water movement across the endothelium. Proper control of stromal hydration is critical, as disturbances in fluid balance can lead to corneal opacity due to edema [6,13,49]. It has also been reported that endothelial injury is associated with reduced AQP1 expression, which may contribute to impaired fluid regulation and subsequent corneal clouding [50].

The corneal endothelium plays a central role in fluid homeostasis through a coordinated mechanism involving both active ion transport and passive water movement. This so-called “pump–leak” system relies on multiple ion transporters, including Na^+^/K^+^-ATPase, Na^+^/K^+^/2Cl^−^ cotransporters, and HCO_3_^−^/Cl^−^ exchangers, which actively move solutes from the stroma into the aqueous humor [5,45,46]. The resulting osmotic gradient drives water movement from the stroma toward the anterior chamber, a process facilitated in part by AQP1 channels and, to a lesser extent, by paracellular pathways [45,46]. Additionally, the transport of metabolites such as bicarbonate and lactate contributes to maintaining this osmotic gradient, further supporting fluid regulation [46,51,52].

Through this integrated system, the endothelium effectively removes excess fluid from the stroma, thereby preventing swelling and preserving corneal clarity and visual function [53,54,55,56]. Beyond its role in fluid balance, the endothelium also contributes to metabolic exchange by facilitating nutrient uptake and waste removal. This is achieved through mechanisms including facilitated diffusion (e.g., glucose transport via GLUT1) and secondary active transport systems, such as lactate/H^+^ and lactate/Na^+^ cotransporters [51].

The strong and uniform expression of AQP1 observed in the corneal endothelium across all regions in this study further supports its fundamental role in maintaining corneal hydration. By enabling efficient water transport from the stroma into the anterior chamber, AQP1 contributes to the prevention of stromal edema and the preservation of corneal transparency, which are essential for normal visual function.

A unique distribution pattern that probably supports the camel’s ability to keep corneal transparency in dry situations is revealed by the accurate mapping of AQP1 using assistive AI tools. We found a high density of AQP1 in the epithelium, stromal keratocytes, and endothelium using machine learning-driven segmentation, indicating a strong fluid transport mechanism required to offset the desert’s high evaporation rates. The Random Forest classifier’s objective quantification guarantees that these observations are not only qualitative but, rather, reflect a major physiological adaptation that enables the camel cornea to stay hydrated and functioning in the face of severe osmotic and temperature stress.

## 5. Conclusions

The consistently high expression of AQP1 across all corneal regions indicates that the camel corneal endothelium possesses a highly efficient mechanism for regulating water transport. This capability is essential for maintaining stromal hydration and preventing corneal edema, particularly under the extreme environmental conditions characteristic of arid habitats. The elevated risk of dehydration in such environments likely necessitates enhanced fluid control to preserve corneal transparency and visual function.

Moreover, the observed variations in corneal layer thickness appear to correspond with regional differences in AQP1 expression. Areas associated with increased metabolic activity or intensified fluid transport demands exhibited more pronounced structural adaptations. Together, these findings suggest a coordinated relationship between corneal morphology and aquaporin distribution, reflecting specialized adaptations that support ocular function in desert conditions.

## Figures and Tables

**Table 1:** Description of basic information about the examined animals.

Stain	Species (Tissue)	Age (Average)	Sex	General Conditions
H&E	6 Corneas	5 years	3 Males 3 Females	Under healthy conditions
IHC	6 Corneas	7 years	3 Males 3 Females	Under healthy conditions

**Table 2:** Description of the Measurements of Corneal Epithelium thickness among different corneal regions databases using assistive AI tools. Mean epithelial thickness ≈ 123.2 µm. Maximum Middle Temporal Corneal Region (137.71 µm). Minimum Central Corneal Region (107.09 µm).

Region	Thickness
C	107.09
MD	126.14
MV	135.03
MN	137.14
MT	137.71 (highest)
PD	111.75
PV	108.36
PN	131.22
PT	114.29

**Table 3:** Description of Measurements of Corneal Stromal thickness among different corneal regions databases using assistive AI tools. Mean stromal thickness ≈ 517.2 µm, thickest regions: middle ventral corneal region, Peripheral Dorsal corneal region and peripheral temporal corneal region, thinnest: middle temporal region.

Region	Thickness
C	433.21
MD	557.78
MV	637.22 (highest)
MN	431.67
MT	378.33 (lowest)
PD	622.22
PV	491.67
PN	491.67
PT	616.67

**Table 4:** Description of Measurements of Corneal Descemet’s Membrane thickness among different corneal regions databases using assistive AI tools. Most corneal regions ≈ 25–30 µm and middle temporal corneal region was dramatically thicker (63.89 µm).

Region	Thickness
C	25
MD	24.44
MV	25
MN	25.56
MT	63.89 (outlier/highest)
PD	25.56
PV	30.56
PN	30.56
PT	42.22

## Data Availability

The original contributions presented in this study are included in the article. Further inquiries can be directed to the corresponding author.

## Abbreviations

The following abbreviations are used in this manuscript:

Abbreviation	Full Name
EPI	Epithelium
BM	Basement membrane
BL	Bowmans Layer
DM	Descemet Membrane
EN	Endothelium
C	Central
MD	Middle Dorsal
MV	Middle Ventral
MN	Middle Nasal
MT	Middle Temporal
PD	Peripheral Dorsal
PV	Peripheral Ventral
PN	Peripheral Nasal
PT	Peripheral Temporal
AQP	Aquaporin
